# Modeling the mechanisms through which conditions of poverty are associated with late language emergence in young children

**DOI:** 10.1016/j.ecresq.2025.07.003

**Published:** 2025-07-24

**Authors:** Britt Singletary, Laura M. Justice, Hui Jiang, Winifred Wilberforce, Daniela Avelar

**Affiliations:** Crane Center for Early Childhood Research & Policy, The Ohio State University, Columbus, OH, USA

**Keywords:** Disrupted parenting, Family Stress Model, Language delay, Late language emergence (LLE), Late talkers, Economic strain

## Abstract

Children growing up in households that undergo economic hardship experience relatively higher rates of language delay and disorder compared to those growing up in homes with more socioeconomic means. The Family Stress Model provides a conceptual model for understanding how experiencing poverty may influence late language emergence (LLE; aka late talking) in young children at age two years. Using a five-dimensional model, we explore the hypothesis that economic hardship and pressure influence parental psychological distress and relationship conflict, in turn leading to disrupted parenting, which may contribute to LLE in young children. We use 14 key indicators to model these five dimensions of the Family Stress Model in our analytical sample of 246 mothers and their two-year-old children experiencing low income. Results provide support for the Family Stress Model as applicable to understanding the contribution of experiencing poverty to LLE, such that the z-score of the likelihood of a child being identified as a late talker was expected to increase by 0.22 with a one standard deviation increase in disrupted parenting. Our analyses suggest a mechanism through which experiences with poverty may disrupt early language development, although further exploration of the impacts of family dynamics within this crucial developmental period is warranted.

## 1. Introduction

**Fig. 1.**
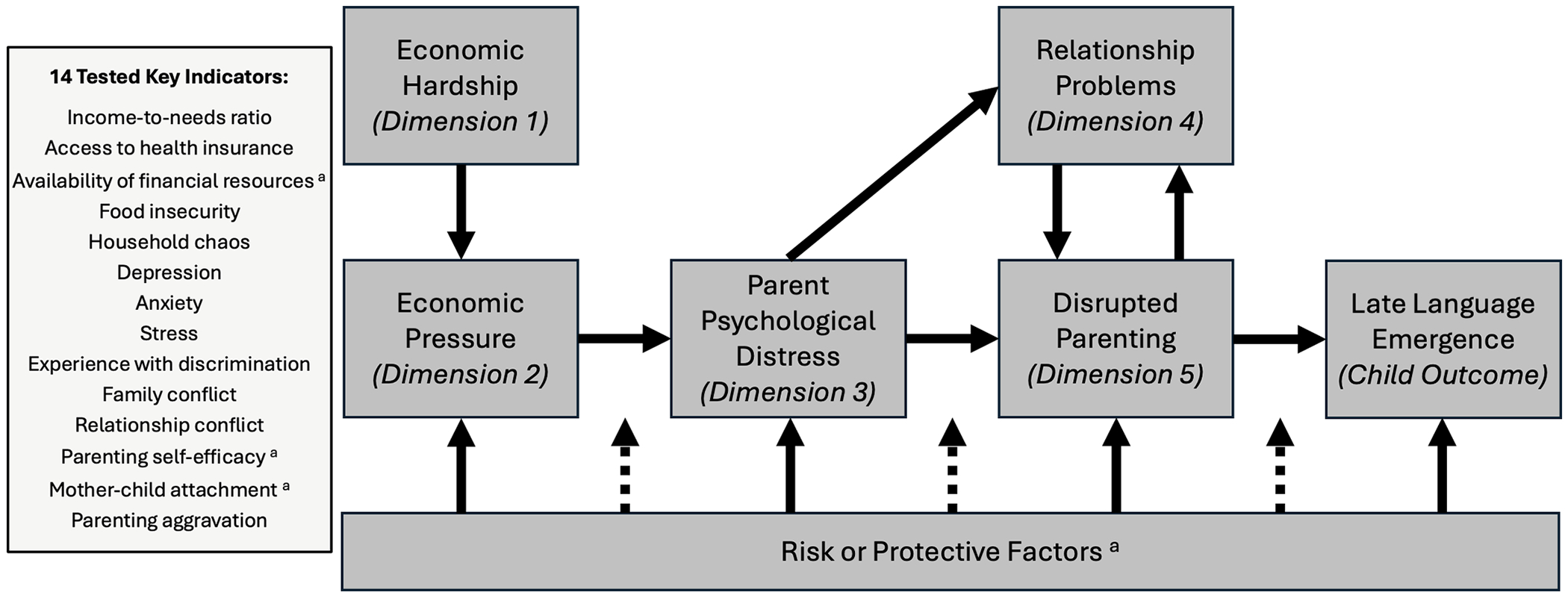
Tested family stress model, as adapted from Masarik & Conger (2017). Note. ^a^While Masarik & Conger (2017) depict risk or protective factors as separate from the five dimensions with these factors acting on a variety of relationships throughout the model (either through main or interactive effects, as shown in the box that runs the length of the model), in the current study we include three potential protective factors (as marked in the 14 Tested Key Indicators box) embedded within the dimensions of the main model, and thus do not test their effects separately.

Nearly 20 % of young children in the United States reside in households near or below the nation’s poverty threshold (Creamer et al., 2022). Considerable research has shown that being reared in households experiencing poverty increases children’s risks for adverse developmental outcomes, including heightened rates of developmental delays and disabilities (Noble et al., 2005; Norbury et al., 2016) and social, behavioral, and mental-health challenges (Bernard et al., 2015; Eamon, 2000, 2001; Tracy et al., 2008). In part, the observed interrelations between household experiences with poverty and children’s developmental outcomes reflect the ways in which economic hardship may disrupt the family context, as theorized in the Family Stress Model, as originally presented by Conger and Conger (2002). In the present study, we sought to evaluate a five-dimensional Family Stress Model to determine the pathways through which experiences with poverty – in the form of economic hardship and pressure – may contribute to children’s language development at two years of age, as illustrated in Fig. 1 (adapted from Masarik & Conger, 2017).

### 1.1. Children’s developmental outcomes: late language emergence

Our model focused on the developmental outcome of children’s susceptibility for late language emergence (LLE), otherwise known as late talking. Children are typically identified as late talkers when they are around 24 months, as this is the age at which most children are producing words and starting to combine words (Rescorla & Dale, 2013); thus, children who are producing few words and no multi-word combinations are often deemed to be late talkers. Presence of LLE in the toddler years can signal heightened susceptibility for future diagnosis of developmental language disorder (DLD), in which children exhibit significant unexplained lags in language development (Walters Jr et al., 2020). Given the heightened susceptibility for DLD among toddlers with delayed language development (Sansavini et al., 2021), early intervention may be warranted to reduce affected children’s risk for future DLD (Cable & Domsch, 2011).

Identification of LLE is possible through use of parent-report instruments in which parents provide input on the number of words their children are producing as well as their use of multi-word combinations (e.g., *that’s my truck*). Commonly used measures to screen for LLE are the MacArthur-Bates Communicative Development Inventories (CDI: Words and Gestures, Words and Sentences, and Checklist; Fenson et al., 1993, 2007; Marchman et al., 2023) and the Language Development Survey (LDS; Rescorla, 1989). Both assessments are available in several different languages, thus permitting comparisons of LLE prevalence in a variety of geographic settings. Studies that use the CDI: Words and Sentences tend to use scores at or below the 10^th^ percentile in productive vocabulary at 24 months of age as the criterion to identify children as late talkers, indicating a prevalence of LLE at this age of between 10 % and 20 % (Bleses & Vach, 2013; Collisson et al., 2016; Desmarais et al., 2008; Horwitz et al., 2003; Korpilahti et al., 2016; Rescorla, 1989). A similar prevalence of late talkers was found in an epidemiological study in Australia using the communication scale of the Ages and Stages Questionnaire (ASQ; Squires et al., 1999); in this study, 13 to 19 % of children were identified as late talkers based on application of various criteria for identifying LLE (Zubrick et al., 2007). Such studies indicate that LLE affects a non-trivial percentage of young children, signaling the need for ongoing research to identify those factors that may contribute to longer-term impairment of language, or DLD, among youngsters with LLE.

### 1.2. Visualizing the effects of poverty on family context

The Family Stress Model (e.g., Conger & Conger, 2002; Masarik & Conger, 2017) may be useful for identifying the potential interplay among experiences with poverty and the family context that can either impede or advance language development among children reared in families experiencing low income (Justice et al., 2019), in part due to the salient stressors within the home environment of the young developing child. For instance, poverty as marked by family hardship and limited resources can deteriorate parents’ mental well-being which, in turn, can increase interfamily conflict and detract from caregivers’ provision of sensitive, responsive interactions with their children (see Cooper & Stewart, 2021 for review), which are important to foster early language development (Ridley et al., 2020). This pathway of economic pressure and hardship → parent psychological distress, interparental relationship problems, and disrupted parenting → children’s language development (see Fig. 1) is derived from the Family Stress Model to depict how experiences with poverty can impede young children’s language development (Masarik & Conger, 2017).

Yet not all families experiencing low-income will experience it in the same ways, and child developmental outcomes from growing up in these contexts vary – children can grow up in low-income households and thrive. Masarik & Conger’s (2017) Family Stress Model leaves room for potential protective factors, like parental social support, effective coping strategies, and/or a sense of optimism, that may moderate the family stress process (see Fig. 1). Thus, despite myriad potential stressors that families experiencing poverty face, individuals facing these stressors vary greatly with how they respond to these conditions according to strengths and assets they may possess as well, which act to buffer and mitigate the downstream effects of economic hardship on children’s developmental outcomes.

Theoretically, we propose that the primary pathway through which experiences with poverty contribute to LLE is indirect, operating through the impacts of economic hardship (dimension 1) and economic pressure (dimension 2) on parent psychological distress (dimension 3) and relationship problems (dimension 4) which, in turn, contribute to disrupted parenting (dimension 5). While we do not assess the main or interactive effects of protective or mitigating factors that could exert pressure on the entire Family Stress Model (see Fig. 1), several of our 14 key indicators which we use to represent these dimensions are positive or strength/asset-based indicators. Within the economic hardship dimension, we include availability of financial resources (a measure of material assets that may help families meet their needs). Within the disrupted parenting dimension, we include parenting self-efficacy and mother-child attachment, which when present in some families as high self-efficacy and secure attachment are a measure of parenting strengths.

### 1.3. Conditions of poverty and experiences of stress

Parents in some families experiencing poverty may be forced to balance tradeoffs between time spent working to make ends meet with time spent with children, also leaving less cognitive bandwidth overall for investing in parenting strategies (Wimer & Wolf, 2020). These trade-offs may also lead to increased parenting stress. While many parents, regardless of socioeconomic status, will occasionally exhibit parenting stress linked to the normal day-to-day hassles of raising children, some individuals may experience more parenting stress than others, particularly if they also experience depression and/or poor social support for example (see Fang et al., 2024 for review). Additionally, there are associations between parenting stress and child-level characteristics during early childhood, such that parents of children with more behavioral problems (for example) may also exhibit more parenting stress (e.g., Fang et al., 2024; Kochanova et al., 2022). Social support may help mediate this association (e.g., Schellinger et al., 2020).

As parenting stress and parenting style may be associated, finding ways to reduce parenting stress *and* intervene on parenting styles (to encourage more supportive and warm parenting approaches versus harsh or controlling approaches) may be an avenue through which to adjust potential impacts on child outcomes like externalizing behavior problems (e.g., Mak et al., 2020). Further, there are associations between parenting stress, depressive symptoms, and marital satisfaction experienced by the mother and father independently within a shared household, suggesting that the inclusion of both parents’ experience with stress is important to more holistically represent stress across the family system and its associations with child behavior problems (e.g., Lim & Shim, 2021; Matalon et al., 2022; Ponnet & Wouters, 2014; Zietz et al., 2022). Longitudinal data across seven countries suggests that economic hardship indirectly effects behavioral outcomes during adolescence particularly through these maternal and paternal experiences within tests of the Family Stress Model (Zietz et al., 2022).

Finally, systemic barriers, including racism and historical trauma beyond the control of the individual family can impact the experience of parenting and psychological distress within the family system for households experiencing poverty (e.g., Sege et al., 2022). For example, parents of households experiencing low-income may face barriers in terms of access to support and intervention services related to both their financial needs and/or their children’s developmental needs (social, emotional, or cognitive). They also face barriers in access to adequate and high-quality medical and diagnostic care, child care, housing, neighborhoods, and/or social support, all of which may increase their experience of parenting stress, which in turn may impact their parenting behaviors (see Cook et al., 2024 for review).

### 1.4. Disruptions to pathways of child language development

In the present study, we test the premise that the Family Stress Model may provide a theoretically plausible means to understand how experiencing poverty within the early family context can contribute to children’s risk for LLE in early childhood. As noted previously, the Family Stress Model proposes that the impacts of poverty, that is, the concrete circumstance of economic hardship, disrupts multiple dimensions of the family context which, in turn, affect children’s developmental outcomes. The Family Stress Model has been tested in relation to a number of adverse child and youth developmental outcomes in the social-emotional domain, such as externalizing and internalizing behavior problems (Conger et al., 2002; Neppl et al., 2016), conduct problems (Shelleby, 2018), and mental health symptomatology (Yoder & Hoyt, 2005); such work has helped to identify specific dimensions of the family context that contribute to adverse outcomes among children and youth.

While Perkins and colleagues (2013) argued that the Family Stress Model represents a viable conceptual model for identifying specific mechanisms through which poverty adversely affects children’s language development. To date, however, studies seeking to establish family-level risk factors for LLE have generally only superficially addressed socioeconomic status as a potential factor (Dale et al., 2014; Zambrana et al., 2014), focusing largely on proxy measures of parent educational attainment and occupation status. An exception is a study that longitudinally evaluated the mediating role of three distinct dimensions of family stressors (parental distress, parent-child dysfunctional interaction, and maternal depression) in influencing 2-year-old children’s language skills within a birth-cohort sample from households experiencing low income (Justice et al., 2019). In this sample of 229 2-year-olds and their mothers, 84 % of households had annual incomes below $30,000, and of these, 57 % had annual incomes less than $10,000. On average, children’s receptive scores were about – 1 standard deviation below the mean on a standardized assessment, and 37 % met criteria for LLE, which is three times greater than prevalence estimates in general populations (e.g., Zubrick et al., 2007). Using path analysis, study findings indicated that household economic hardship and pressure was associated with children’s language skill at 2 years through the mediating pathway of parental distress and parent-child dysfunctional interaction (Justice et al., 2019). This work did not explore linkages between these family stressors and children’s risk for LLE (rather, children’s language was included as a continuous variable), yet the findings help to pinpoint dimensions of the family context that may link experiences with poverty to early adverse language outcomes.

Currently, there is keen interest in both documenting and understanding the influence of experiences with poverty on young children’s language development. Evidence indicates that experiencing poverty can negatively affect cortical brain regions supporting language functions (Noble, Engelhardt, et al., 2015, 2015), which may explain why young children growing up in low-income households tend to perform lower than affluent peers on normative measures of language skill (Fernald et al., 2013; Karwath et al., 2023) and are overrepresented among children with DLD (Norbury et al., 2016). To date, however, there has been less attention directed towards understanding the specific pathways through which early childhood experiences with poverty, a circumstance in which families have diminished economic resources, may disrupt early language development and lead to heightened risk for LLE in young children.

If the experience of living in poverty disrupts parent-child interactions (e.g., NICHD-Early Child Care Research Network, 2005), this may in turn influence young children’s language-learning processes, leading to the observed discrepancies in early language processing reported in young children growing up experiencing low income (e.g., Fernald et al., 2013). During infancy and toddlerhood, when children spend most of their time at home, children acquire language through exposure to abundant and high-quality language input, including conversational turn-taking and a rich variety of vocabulary and grammar with their parents (e.g., Huttenlocher et al., 2010; Rowe, 2012; Weizman & Snow, 2001).

Some parents of households experiencing poverty may engage in lower quantity and quality linguistic interactions with their children, perhaps due to increased stressors, such as depression, faced by these parents (e.g., Hirsh-Pasek et al., 2015; Hoff, 2003; Pan et al., 2005; Rowe, 2008). Consequently, experiencing low income within the household may increase the risk of LLE in young children potentially through changes to language quality and quantity experienced between parent and child (e.g., Horwitz et al., 2003; Zubrick et al., 2007). While research has shown that higher parental stress can be detrimental to children’s language skills (e.g., Noel et al., 2008), a more recent study with families experiencing low income revealed that maternal psychological stress, rather than environmental stressors, was more strongly related to children’s language scores (Troller-Renfree et al., 2022). Maternal stress might change or impact the quantity and quality of language children are exposed to at home which in turn affects children’s language development.

### 1.5. The current study

The purpose of this study was to test the applicability of a five-dimensional Family Stress Model to understand the mechanisms through which poverty, as indexed by economic hardship and economic pressure, may contribute to LLE among two-year-old children through downstream effects on the family system. Specifically, we tested the hypothesis that economic pressure and hardship (dimensions 1 and 2, respectively) → parent psychological distress (dimension 3), interparental relationship problems (dimension 4), and disrupted parenting (dimension 5) → children’s risk for LLE, as indicated by child late talker status at around 2 years of age within a sample of households experiencing low income (Fig. 1).

## 2. Methods

### 2.1. Study participants

Participants in the present study were enrolled in the *SMALL Talk: A Study of Milestones to Advance Language Learning* longitudinal study (*N* = 356). Mother-child dyads were recruited during a primary enrollment period between October 2019 and December 2020 (*n* = 337) and a replenishment period between August and November 2021 (*n* = 19). During the primary period, mothers were initially recruited in-person at local events and centers serving families experiencing low-income in central Ohio (*n* = 20 mother-infant dyads). Following the onset of the COVID-19 Pandemic in March 2020, recruitment moved to remote text advertisements sent by Women, Infant, and Children (WIC) centers to their eligible families in central Ohio, resulting in the enrollment of an additional 317 mother-infant dyads. During the second year of the study, the replenishment period was also conducted via remote text advertisements to WIC-eligible families; 19 additional mother-infant dyads were enrolled to account for attrition during the first year of the study.

Prior to enrolling in the study, all mothers were asked to complete an eligibility screener before being invited to consent to participate. To ensure our sample was representative of households experiencing low incomes, mothers must have either indicated that they resided in households <200 % of the 2019 federal poverty level for their household size (i.e., mothers were asked their annual income and household size, and this was used to calculate their relation to the federal poverty level) or that they had been currently receiving governmental assistance (e.g., WIC, food stamps, CareSource, Medicaid, or housing subsidies). Additionally, mothers were asked to ensure that they were: ≥ 18 years of age, comfortable reading and speaking in English (even if they also spoke (a) language(s) other than English, as English was the only language used for communicating and collecting data with families in this study) and had no impending plans to move out of the general area. Finally, mothers were asked to ensure that their focal children: (a) were from singleton births of 35 weeks or greater gestation (to control for the potential effects of multiple gestation or pre-term birth on language outcomes (e.g., Fasolo et al., 2010; Foran et al., 2021; Foster-Cohen et al., 2007; Guarini et al., 2009; Rand et al., 2005; Sutcliffe & Derom, 2006), (b) had no diagnoses of significant disability at or around birth (to control for the potential effects of early disability diagnoses on language outcomes (e.g., Boyle et al., 1994; Grizzle & Simms, 2005; Shonkoff et al., 1992), and (c) were between 6 and 12 months old (during the primary period) or 13-15 months old (during the replenishment period, who joined in the second timepoint of the study) at the time of enrollment.

### 2.2. Study procedure

Data for the current study were collected across four consecutive timepoints between January 2020 and December 2022, with each timepoint occurring about three to six months apart for each participant, based on the child’s age and mother’s scheduling availability. While only mothers enrolled during the primary period completed the first timepoint (*n* = 337), all mothers (including those enrolled during the replenishment period, *n* = 19) were asked to participate in subsequent timepoints. At each timepoint, mothers were given gift cards following completion of all questionnaires. This study was approved by the authors’ Institutional Review Board.

Between January 2020 and March 2021, timepoint 1 data (hereafter TP1) were collected when children were ~ 6-12 months old from 337 mothers. Prior to March 2020, data were collected using a combination of self-reported and interview-based questionnaires using an iPad hosting Qualtrics secure software application, administered with a trained assessor while visiting participants’ homes (*n* = 20). From April 2020 forward, due to the COVID-19 Pandemic, we transitioned to remote data collection, which included a phone call with a trained assessor to conduct interview-based questionnaires, followed by sending the mother more sensitive self-report questionnaires to complete on her own via personalized Qualtrics links sent by email (*n* = 317). Not all mothers completed all emailed survey links (*n* = 13 did not finish), such that while interview-based questionnaires were completed by all 337 mothers as administered by trained assessors, there is variation in sample sizes for self-report measures administered by email.

Timepoint 2 data (hereafter TP2) were collected when children were ~ 13-15 months old from 305 mothers. All data were collected via interview-based questionnaires by phone and self-report questionnaires by email. As previously, following the phone call (*n* = 305), not all mothers completed all emailed survey links (*n* = 19 did not finish), such that there is variation in sample sizes for self-reported data.

Timepoint 3 data (hereafter TP3) were collected from 268 mothers when children were ~ 19-21 months old. All data were collected via interview-based questionnaires by phone and self-report questionnaires by email. As previously, following the phone call (*n* = 268), not all mothers completed all emailed survey links (*n* = 30 did not finish), such that there is variation in sample sizes for self-reported data.

Timepoint 4 data (hereafter TP4) were collected when children were ~ 24-27 months old from 246 mothers. All data were collected via interview-based questionnaires by phone and self-report questionnaires by email. As previously, following the phone call (*n* = 246), not all mothers completed all emailed survey links (*n* = 26 did not finish), such that there is variation in sample sizes for self-reported data.

### 2.3. Measures

For the current analyses, we use data from TP1 through TP4, administered via either interview or emailed surveys (as indicated below). Interview-based surveys took 30-60 minutes to complete at each timepoint, while the corresponding emailed surveys took participants another 30-60 minutes to complete on their own at their convenience in the weeks following the interview-based surveys at each timepoint.

To assess the potential pathway through which family stress may be associated with LLE, we used measures to represent: (1) 14 key indicators encompassing the five dimensions of the Family Stress Model, (2) child language skills and LLE status, and (3) key covariates (i.e., child age, gender, and home language).

#### 2.3.1. Family Stress Model

The Family Stress Model comprises five dimensions – economic hardship, economic pressure, parent psychological distress, relationship problems, and disrupted parenting. These dimensions are represented by 14 key indicator variables, carefully selected to provide a holistic picture of the various stressors families experiencing low income may face. Here we provide a summary of the measures used to represent these dimensions, while further details of the measures used are listed in Table 1.

The dimension of *Economic Hardship*, here defined as the financial constraints experienced by a family, included three key indicator variables: income-to-needs ratio and access to health insurance (both assessor-led at TP1 and TP2), as well as availability of material financial resources (emailed at TP1 and TP2). The dimension of *Economic Pressure*, here defined as the psychological and emotional circumstances that result from experiencing hardship, included two key indicator variables: food insecurity and household chaos insecurity, both collected by email at TP1 and TP2. The dimension of *Parent Psychological Distress*, here defined as the mother’s emotional well-being, included four key indicator variables: depression (emailed at TP1), anxiety (emailed at TP1 and TP2), stress (emailed at TP2), and experience with discrimination (emailed at TP2). The dimension of *Relationship Problems*, here defined as conflict within the family, included two key indicator variables: family conflict and relationship conflict (both emailed at TP1 and TP2). The dimension of *Disrupted Parenting*, here defined as the disturbance of the mother-child relationship, included three key indicator variables: parenting self-efficacy, mother-child attachment, and parenting aggravation (all emailed at TP1 and TP2).

To ensure high validity and reliability in measuring each construct, for scaled indicators (12 out of 14 indicators) we used previously published measurement scales to collect data to ensure high validity in measuring each construct. In other instances (i.e., two indicators of economic hardship), the responses to single items represent key indicators (e.g., income-to-needs ratio and access to health insurance. For scaled indicators, internal consistency was calculated across our analytical sample to test measure reliability, such that a range is provided if the measure was collected at multiple timepoints.

#### 2.3.2. Child language skills and LLE status

Child language skills were measured via parent-report using the Communicative Development Inventories (CDI; Fenson et al., 1993, 2007; Marchman et al., 2023), specifically the Words and Gestures form (CDI-WG) administered at TP2 (normed and validated for children aged 8-18 months, most recent edition: Marchman et al., 2023), and the Words and Sentences form (CDI-WS) administered at TP3 and TP4 (normed and validated for children aged 16-30 months, most recent edition: Marchman et al., 2023). The CDI-WG consists of a total of 429 questions, including a list of 396 vocabulary words that the child may be able to say. The CDI-WS, consists of a total of 796 questions, including a list of 680 vocabulary words that the child may be able to say.

In the current study, we focus on only the vocabulary sections of the CDI-WG and CDI-WS, and the resulting Total Words Produced scores from each, as it is a common practice to use productive vocabulary (as measured by total words produced) for identifying later talkers using these particular measures (e.g., Bleses & Vach, 2013; Collisson et al., 2016; Desmarais et al., 2008; Horwitz et al., 2003; Korpilahti et al., 2016; Rescorla, 1989). In each form, the mother is asked to indicate whether the child can say each listed word in English but might instead select options for *I don’t know* or *Prefer not to answer* for each listed word, which resulted in the potential for some missing data for each participant. Thus, a sum score was calculated for participants who answered at least 317 items on the CDI-WG and 544 items on the CDI-WS, respectively, such that we included data for reports with <20 % total missing data across all vocabulary words. Subsequently, the Total Words Produced score for each child was converted to a percentile rank score referencing a normative sample adjusted by gender and age (using the 3rd edition; Marchman et al., 2023) to produce the primary measure of interest, namely the percentile rank that denotes the relative positioning of the children’s language skills within the comparable population. Consistent with previous studies, we utilized the 10th percentile as the threshold for designating late talker status, such that children scoring at or below the 10th percentile in CDI-WS were identified as late talkers, whereas those above this rank were categorized as non-late talkers (e.g., Avelar et al., 2025; Bleses & Vach, 2013; Collisson et al., 2016; Desmarais et al., 2008; Horwitz et al., 2003; Korpilahti et al., 2016; Rescorla, 1989).

#### 2.3.3. Covariates

Several covariates were included in our analysis: child’s gender (male or female), child age at test (in months), and primary home language (English or other). Each of these variables has been shown to be related with measures of child language skills during this early stage of development (e.g., Buac et al., 2014; Fenson et al., 1994; Huttenlocher et al., 1991; Quiroz et al., 2010). Child’s gender and home language were measured at enrollment. Child’s age at test was calculated using the child’s date of birth and the completion date for the CDI survey, which was based on the timestamp from the respective parent-report survey form.

### 2.4. Analytical overview

**Fig. 2.**
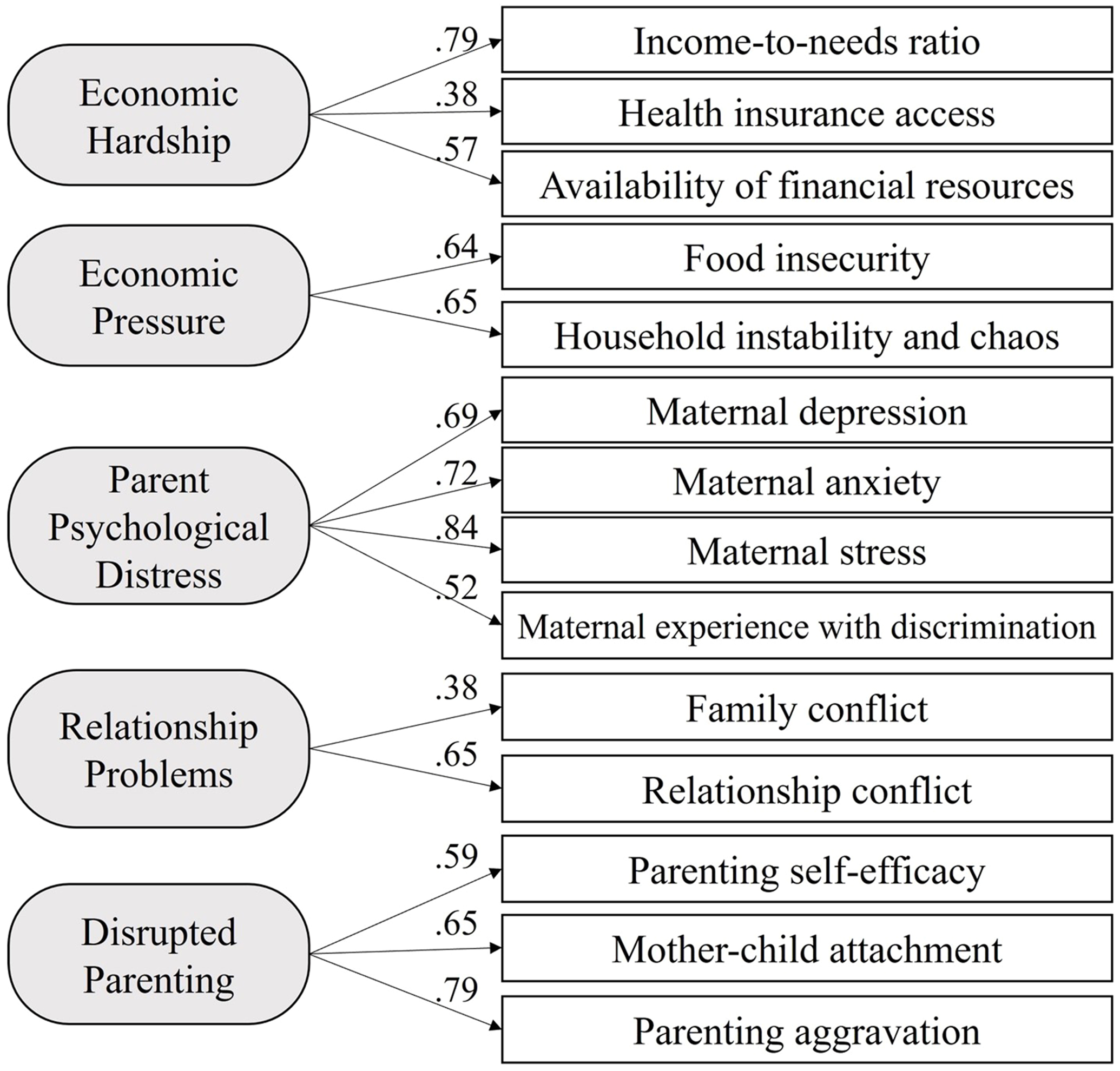
Family stress model: Confirmatory factor analysis, including factor loadings (listed to the left of each key indicator). Note. All indicators were collected from TP1 and TP2.

Based on our analytical sample of 246 participants with valid language assessment data from at least one timepoint, we evaluated the relationship between family stressors and children’s language development from age ~ 1.5 years (TP3) to ~ 2 years (TP4). In an initial analysis, we validated the five-dimensional Family Stress Model (see Fig. 1; adapted from Masarik & Conger, 2017) via Confirmatory Factor Analysis (CFA), thereby deriving factor scores representing each of the five dimensions of family stressors: Economic Hardship, Economic Pressure, Parent Psychological Distress, Relationship Problems, and Disrupted Parenting (Fig. 2). We then examined descriptive statistics of the key variables, including the five indicators of earlier family stressors (at TP1 and TP2) and later child language skills measured (at TP3 and TP4). Additionally, we calculated pairwise correlation indices, using Pearson correlation coefficients to measure the magnitude of correlation between continuous or binary indicators, and Spearman correlation coefficients in cases where at least one indicator was ordinal or exhibited an extremely skewed distribution.

We then conducted path analysis to investigate the relationship between family stressors and LLE status, using Mplus 8.0 (Muthén & Muthén, 2012). Building upon the framework proposed by Masarik and Conger (2017), we hypothesized that Economic Hardship precipitated Economic Pressure, subsequently leading to Psychological Distress in mothers. This distress, in turn, could lead to Disrupted Parenting and Relationship Problems, and ultimately Disrupted Parenting could inversely impact early language development, demonstrated by a higher likelihood of the child being identified as a late talker between 1.5 (TP3) and 2 years of age (TP4).

**Fig. 3.**
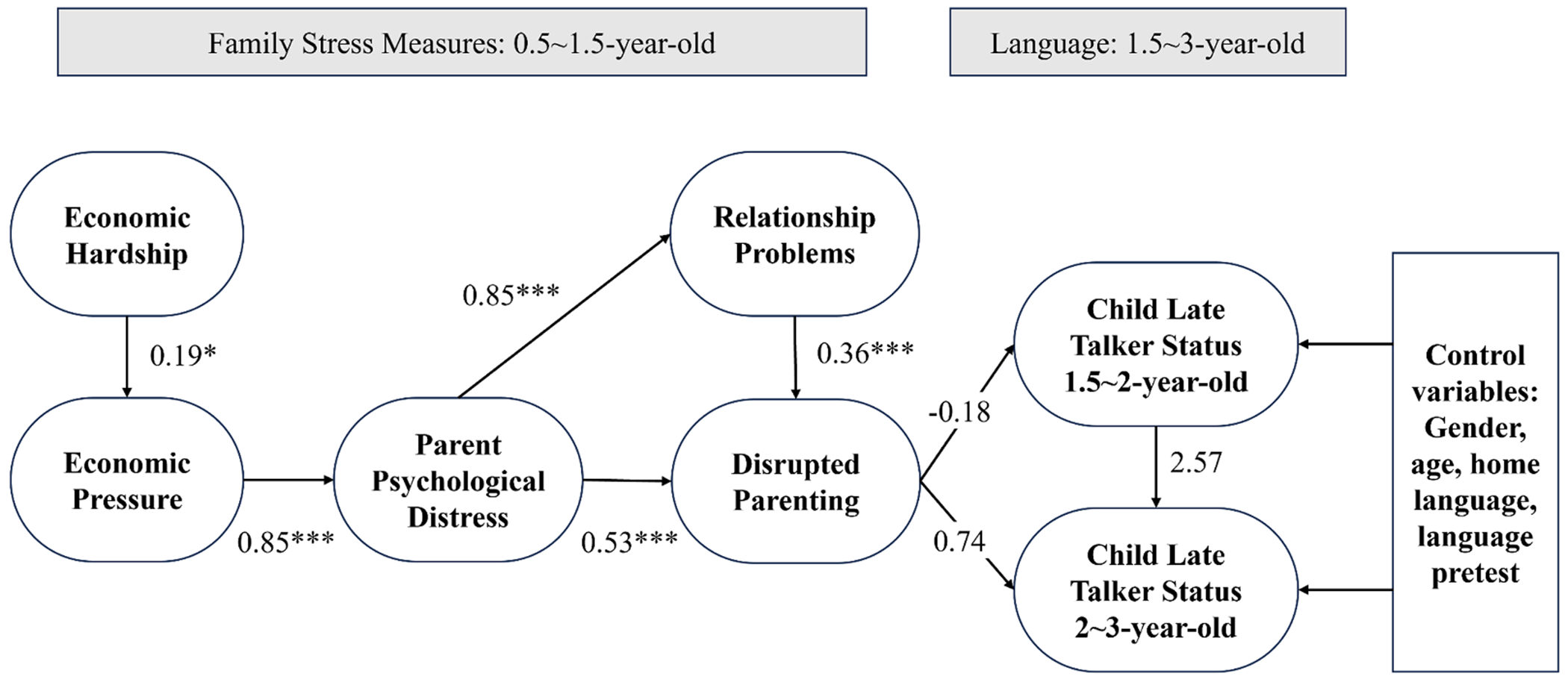
Family stress model and children’s early language disruption. Note. The numbers represent unstandardized coefficients of the paths. All continuous variables were standardized to have a mean of 0 and standard deviation of 1. *** *p* < .001.

We tested the model depicted in Fig. 3, estimating the inter-relationships among key variables concurrently using weighted least squares, mean and variance adjusted (WLSMV; Muthén, 1984). Controlling for covariates including child gender, age at test, and home language assessed at around age 1 year (TP2), linear regression was used for modeling other paths, whereas probit regression was used to model children’s late talker status, a binary outcome variable. The probit regression links the inverse standard normal distribution of the probability (i.e., z-score of probability) to a linear combination of predictors. Evaluation of the overall model fit involved the chi-square test of model fit statistic, root mean square error of approximation (RMSEA), comparative fit index (CFI), and Tucker-Lewis index (TFI) (Hu & Bentler, 1999).

Within the analytical sample, there were no missing data for Family Stress Model measures. However, missing data ranged between 6 % to 24 % for outcome measures and from 0 % to 24 % for covariates. When estimating the path models, we employed the full information maximum likelihood (FIML) technique to treat missing outcome variable data (Arbuckle et al., 1996).

## 3. Results

### 3.1. Validation of the Family Stress Model

We conducted confirmatory factor analysis to validate the five-dimensional Family Stress Model (Fig. 2) and derive factor scores to measure Economic Hardship, Economic Pressure, Parent Psychological Distress, Relationship Problems, and Disrupted Parenting. Factor loadings obtained from CFA ranged from .38 to .84, and all model fit indices indicated an adequate fit of the model to the data: RMSEA =.037, with a 95 % confidence interval of .020 to .052; CFI =.959; and TLI =.943. The Chi-square value was 97.65 (degree of freedom = 66, *p* =.007). Factor scores extracted from the model were standardized for use in subsequent analyses.

### 3.2. Descriptives of language development and LLE status

As presented in Table 2, mothers provided CDI – Total Words Produced data for their children at three timepoints to measure their early language skills. On average, the scores for sampled children corresponded to the 45^th^ percentile (TP2), 34^th^ percentile (TP3), and 36^th^ percentile (TP4). This trend reveals a widening disparity in early language development between the sampled children and their same-aged counterparts from the general population (as compared to normative data including individuals across diverse socioeconomic statuses Marchman et al., 2023).

Notably, a substantial proportion of the sampled children – approximately 30 % - met criteria for LLE between ages of 1.5 to 3 years, based on CDI scores falling at or below the threshold for late talker status (10^th^ percentile), indicating high prevalence of delayed language acquisition within our sample.

### 3.3. Correlation between family stressors and LLE status

Table 3 summarizes the correlation coefficients among the five Family Stress Model dimensions, LLE status, and the covariates, and a few notes are warranted. First, Economic Pressure, Parent Psychological Distress, Relationship Problems and Disrupted Parenting exhibited strong inter-correlations, ranging from *r* =.67 to *r* =.87. In contrast, Economic Hardship showed a moderate correlation solely with Economic Pressure (*r* =.31) but not with the other dimensions of family stress. Second, LLE status was highly correlated between the two consecutive TP3 and TP4 (*r* =.66), suggesting continuity in language development across these sequential assessments. However, relatively low raw correlations were found between LLE status and family stressors. Third, the covariates showed varied association with LLE status, ranging from small- to moderate-sized correlations. These covariates were used as control variables in the subsequent path analysis. Table 4 listed a variety of attributes that were used in path analyses and compared them across LLE status at TP4.

### 3.4. Path analysis exploring relationships between family stressors and LLE status

We conducted a path model investigating the relations between dimensions of the Family Stress Model and children’s LLE status at age 1.5 to 3 (Fig. 3). The goodness of fit statistics for the model was adequate: X ^2^ (35) = 37.731, CFI= 0.992; TLI = 0.989; RMSEA = 0.022. Between the Family Stress Model dimensions, we found significant relationships between all theoretical paths. Economic Hardship significantly predicted Economic Pressure (*β* = 0.19, *p* < 0.05). Economic Pressure significantly predicted Parent Psychological Distress (*β* = 0.85, *p* < 0.001), which significantly predicted Disrupted Parenting (*β* = 0.53, *p* < 0.001), and Relationship Problems (*β* = 0.85, *p* < 0.001). Moreover, Relationship Problems significantly predicted Disrupted Parenting (*β* = 0.36, *p* < 0.001). The observed relationship supports our hypothesis that economic conditions and household pressure predict caregivers’ psychological states, which further predicts family relationships and interactions.

As shown in Table 5 and Fig. 3, after controlling for child’s gender, age at test, home language, prior language skills, and Disrupted Parenting (measured at TP1 and TP2) performed in the expected direction in predicting children’s LLE status at TP4, when children were about two years of age (*β* = 0.74, standardized coefficient = 0.22, *p* = 0.241). Although the test statistic does not meet criteria for statistical significance, consideration of the effect-size estimate may indicate the practical import of the findings. Thus, to interpret this effect, for each one standard deviation increase in Disrupted Parenting, the z-score of the likelihood of a child being identified as a late talker was expected to increase by 0.22. This corresponds to an average marginal probability effect of 0.06, or a 6 % point increase in the probability of being identified as a late talker. This corresponds to a moderate effect size considering the base rate of late talkers in this sample is approximately 30 %. Considering that our current sample size limited the power to 0.58 to detect this effect, we believe that practical significance as measured by effect size is informative despite the lack of statistical significance. This observed relationship aligns with our hypothesis, suggesting that Disrupted Parenting when children are young is associated with an elevated risk of LLE.

## 4. Discussion

In the present paper, we sought to test the applicability of the Family Stress Model (Conger et al., 2002) as an integrative theoretical model for representing the mechanisms through which experiences with poverty may influence young children’s language development and, in particular, their risk for LLE. The Family Stress Model proposes that poverty, in the form of economic hardship and the pressure it creates, leads to heightened parent psychological distress, interparental relationship problems, and disrupted parenting, which in turn impedes children’s language development. Consistent with perspectives from neuroscience literature (Noble, Houston, et al., 2015; Noble et al., 2005), the Family Stress Model identifies a variety of mechanisms through which the child experiences heightened stress (e.g., due to increase parent psychological distress and interpersonal relationship problems) and a diminished linguistic environment (e.g., through disrupted parenting). Importantly, the Family Stress Model is multidimensional in nature, identifying a variety of ways in which economic hardship disrupts the family context.

The present study is the first to our knowledge to have tested the Family Stress Model’s applicability for identifying pathways through which experiences with poverty lead to LLE, although this theoretical framework was originally presented, but not tested, by Perkins and colleagues (2013). The present study involved 246 mother-child dyads, all of whom were experiencing low incomes, allowing us to evaluate the Family Stress Model within a cohort of families all experiencing poverty. The present study had three main findings, each of which we discuss in turn.

First, the Family Stress Model appears applicable to understanding the pathways through which experiences with poverty lead to LLE, as presented in Table 2. To our knowledge, this is the first investigation of the Family Stress Model’s applicability to children’s late-talker status, complementing prior studies that have examined such child and youth outcomes as behavior problems (Conger et al., 2002; Neppl et al., 2016; Shelleby, 2018), academic achievement (Benner & Crosnoe, 2011), physical well-being (McCurdy et al., 2010), and school readiness (Iruka et al., 2012). Concerning the latter, scholars used path analysis to explore family processes as mediators between hardship and pressure measures and children’s readiness skills, to include receptive and expressive language, finding that a measure of sensitive parenting mediated the links between caregiver education, a measure of socioeconomic status, and children’s language outcomes at age four years (Iruka et al., 2012).

Iruka and colleagues’ (2012) work confirms the applicability of the Family Stress Model to identifying pathways through which experiences with poverty affects preschoolers’ language development, offering an important framework for the present study. In the present study, we found that the Family Stress Model provides a theoretically and scientifically viable explanation of the ways in which experiences with poverty disrupt the family context and contribute to children’s language outcomes at two years of age. Building upon evidence from the neuroscience literature (Noble, Engelhardt, et al., 2015, 2015; Noble et al., 2005), the Family Stress Model allows for heightened stress and a diminished linguistic environment to serve as contextual factors linking family hardship to children’s language development.

Second, the primary pathway through which the family context contributes to LLE within our sample is through disrupted parenting. Our study supports the presence of this relationship when using a latent variable formed from the three key indicator variables of parent-reported measures of parenting self-efficacy, parental stress, and mother-child attachment to represent the construct of Disrupted Parenting, suggesting that even the mother’s self-perception of her own parenting (as opposed to ratings achieved through direct-observation) is predictive of child outcomes. Disrupted Parenting was associated with a 6 % increase in probability of being identified as a late talker, corresponding to a moderate effect size, suggesting practical significance.

Previous studies have found similar relationships between parenting quality and child outcomes when investigating the Family Stress Model across culturally and socioeconomically diverse groups. For example Iruka and colleagues (2012) used post-hoc coding of mother-child interactions within a large sample (*n* = 9,500) of culturally and socioeconomically diverse children, and found parenting sensitivity to be a salient mediator between family demographics and child outcomes within their tested Family Stress Model, specifically in European Americans, African Americans, and Asian Americans, but not English-speaking Hispanics; meanwhile, negative parenting was only a salient mediator for European Americans only. Additionally, Shin and McCoy (2022) found support for the role of maternal self-efficacy and knowledge of child development in predicting children’s language development in a large sample (*n* = 1,894) of socioeconomically diverse Korean children living in South Korea. Thus, our finding replicates this relationship within a sample of families experiencing low incomes, and finds that cultural group (here evaluated as race and ethnicity) does not impact the predictiveness of this model. Notably, these results suggest an area that can be potentially intervened upon in the future, through interventions that target positive parenting practices and the construction of improved maternal self-efficacy within communities experiencing low incomes.

Additionally, the model may be improved by the inclusion of additional strengths and assets that families experiencing low income may possess, which act to buffer and mitigate the downstream effects of economic hardship on developmental outcomes. Assets can be financial, such as savings and checking accounts, certificates of deposit, money market funds, government bonds, treasury bills, vehicles, and/or properties that can be liquidated to help cover economic needs and mediate the experience of economic hardship when experiencing low income (e.g., Chen et al., 2024, 2023). Alternatively, assets can be experiential. This may include experiences with programs like Early Head Start and Head Start which can act to intervene and promote positive partnerships between parents and the resources they need to improve the lives of their children (e.g., Beckmann, 2017), or community-level initiatives to identify and reduce experiences of maternal depression in families experiencing poverty (e.g., Knitzer et al., 2008). Additionally, a quickly growing research field over the past decade suggests that individual-level exposure to positive childhood experiences (PCEs), also called counter-ACEs (where ACE, stands for adverse childhood events), can help individuals have more resilience in the face of adversity and stress, thus acting as protective factors for long-term developmental outcomes (e.g., Bethell et al., 2019; Burstein et al., 2021; Crandall et al., 2019; Guo et al., 2022; Han et al., 2023; Longhi et al., 2021; Narayan et al., 2023; Raghunathan et al., 2024; Scholtes & Cederbaum, 2024; Sege et al., 2022; Sege & Browne, 2017; Williams, 2023).

In part led by the HOPE (healthy outcomes from positive experiences) framework (Burstein et al., 2021; Sege & Browne, 2017), researchers have begun to investigate how a) positive interpersonal relationships (within family and with peers and non-familial adults), b) equitable, safe, and stable environments, c) a sense of belonging and connectedness, and d) social and emotional growth during childhood act as protective factors mitigating the effects of chronic stress and adversity, like that individuals might experience via poverty (e.g., Bethell et al., 2019), including via the promotion of HOPE-informed prevention strategies to help families better understand how to support children in developing under stressful conditions (e.g., Sege et al., 2022; Sege & Browne, 2017). For example, in a study of 1,169 adolescents (12-17 year-olds), participants’ reported experience with PCEs (in terms of frequency across 7 items) explained about two times the variance in participants’ reported mental health symptoms as compared to their experience with ACEs (1= yes to any one or more studied ACEs versus 0 = none) (Scholtes & Cederbaum, 2024). In another study of 3, 111 children from birth to 15 years, both PCE type and total (by wave and cumulatively) were associated with fewer child mental health problems and fewer academic difficulties over time (Guo et al., 2022). Additionally, higher accounts of PCEs recounted by adults are associated with better physical health indicators and health behaviors, more positive psychosocial functioning and reduced stress, and potentially with higher executive functioning and some positive parenting behaviors (see Han et al., 2023 for review). Taken together, this research suggests that positive experiences that children experience should not be overlooked when trying to understand developmental outcomes in the face of adversity and stress.

Third, our findings here suggest that LLE is over-represented among young children experiencing poverty. Previous literature indicates a prevalence of LLE within the general population at the age of ~24 months at between 10 % and 20 % (Bleses & Vach, 2013; Collisson et al., 2016; Desmarais et al., 2008; Horwitz et al., 2003; Korpilahti et al., 2016; Rescorla, 1989; Zubrick et al., 2007). In the present study, approximately 30 % of sampled children meet criteria for LLE, indicating high prevalence of delayed language acquisition within our sample. This prevalence rate is in-line with previous estimates generated in sample experiencing low incomes within a similar geographic area (Justice et al., 2019), providing further evidence for this over-representation of LLE within families experiencing poverty which is of concern since children with LLE have heightened susceptibility for later diagnosis of DLD (Sansavini et al., 2021).

Epidemiological research finds that children growing up in low-income households are disproportionately affected by DLD (Norbury et al., 2016), signaling the need to understand the mechanisms through which experiences with poverty disrupts children’s language development and creates susceptibility for LLE and, in turn, DLD. Being reared in the conditions of poverty may exert negative effects on young children’s language development, leading to heightened susceptibility for early language delays and longer-term language and learning disabilities (Fazio et al., 1996; Justice et al., 2019; Lindsay & Strand, 2016). As an example, an analysis of the standardized vocabulary scores of children attending needs-based preschool programs found that children performed, on average, nearly one standard deviation below the age-normative range (Cabell et al., 2011). There is increasing evidence that adverse socioeconomic conditions influence early brain development, to include influencing the development of language-supporting neural pathways (Noble, Engelhardt, et al., 2015), with distinct neural differences differentiating advantaged and disadvantaged children (Ursache & Noble, 2016). Two primary mechanisms proposed in the neuroscience literature for explaining linkages between socioeconomic conditions and young children’s brain development are stress and the linguistic environment (Ursache & Noble, 2016). Experiences with poverty can lead to heightened ongoing stress, which can influence several brain areas critical for language development, including the prefrontal cortex. Likewise, experiencing poverty can also reduce caregivers’ capacity to provide high-quality linguistic input, which is a critical mechanism for stimulating early language skills (e.g., Rowe, 2012). Together, the experience of poverty can heighten the child’s ongoing exposure to stress and diminish the child’s linguistic environment, with negative repercussions to the child’s developing language skills.

Although there are many ways in which parenting influences children’s early language development, of particular importance is the role of sensitive and responsive interactions between caregiver and child as well as the quality and quantity of linguistic input. Concerning the former, sensitive and responsive interactions are those in which the caregiver is attuned to the child and provides contingent responses to the child’s verbal and nonverbal communications, which helps to maintain periods of joint attention and back-and-forth communications. A meta-analysis involving 36 samples and 7,315 caregiver-child dyads, with children between 16 and 71 months of age, reported that the relationship between sensitive and responsive caregiver interactions and children’s language skills was significant (*r* = 0.27 [95 % CI: 0.21 to 33] and not affected by publication bias (Madigan et al., 2019). Importantly, moderator analyses showed that these relations were moderated by household socioeconomic status, such that the relations were stronger for lower-income dyads (*r* = 0.37; 95 % CI: 0.19 to 0.53) as compared to more advantaged dyads (*r* = 0.15; 95 % CI: 0.05 to 0.24). Thus, for children residing in households with low income, access to sensitive and responsive interactions with their caregivers may provide an important buffer against the effects of economic hardship and pressure.

Concerning the latter, considerable evidence indicates that the quality and quantity of linguistic input from caregivers to their children is also influential to early language development. The quality of caregiver input refers to the diversity of caregiver talk in terms of the number of different words and multi-clause utterances used, whereas the quantity of input refers to the sheer volume of talk in terms of number of words and utterances (Anderson et al., 2021). Drawing once again from a meta-analysis (Anderson et al., 2021), study findings showed a significant, positive relationship between caregiver input quality and children’s language skills on the basis of 35 samples and 1,958 dyads (*r* = 0.33; 95 % CI: 0.24 to 0.42), with no evidence of publication bias. Additionally, moderator analyses showed that household socioeconomic status was a moderating factor, such that the relations were stronger for lower-income dyads (*r* = 0.39; 95 % CI: 0.34 to 0.52) as compared to middle- and upper-income dyads (*r* = 0.31; 95 % CI: 0.19 to 0.42). Similar findings were reported for caregiver input quantity and its relation to child language skills, for 33 samples and 1,411 dyads (*r* = 0.20; 95 % CI: 0.13 to 0.27). For input quantity, the strength of this relation was not associated with lower-income versus middle/upper-income status.

Notably, as early identification of LLE is tied to later developmental language delays as children age (e.g., Sansavini et al., 2021; Walters Jr et al., 2020), this finding points toward a heightened need to find ways to intervene when children are young in order to reduce the heightened prevalence of LLE related to growing up in households facing economic hardship. As suggested by our study, teaching parents positive and developmentally appropriate ways to interact with their children at an early age may represent a path forward to reducing the incidence of LLE in these populations and thereby possible reduce the incidence of DLD.

### 4.1. Limitations

We point out several limitations and how these can be addressed in future research. First, this study uses a convenience sample, thus, these results cannot be generalized to a larger population. Additionally, as this sample was recruited via advertisements through WIC specifically, it is possible that our resulting sample fails to include the full range of variability of conditions and experiences present in households experiencing low incomes, as we may not have reached families who had more trouble obtaining resources to supplement their needs, such as WIC. In fact, our analyses revealed that there was substantial variation present in the experiences and conditions of poverty among our recruited families. Furthermore, by excluding mothers who were not comfortable speaking and reading English in this study (our only language used in communicating and collecting data with families), our sample does not represent the potentially varied experiences of families who are either non-English speaking or English-as-a-Second-Language users. Finally, as our study excluded children who were born prematurely, from multiple births, or with significant disabilities diagnosed at or around the time of birth, we also cannot investigate how any of these child-level features may influence how the Family Stress Model explains pathways from economic strain to child outcomes. In future studies, a larger sample recruited using more diverse methods and with fewer exclusionary criteria may incapsulate the variability of households experiencing low income more fully.

Second, our model generally focuses on experiences of stress and strain within the family system, rather than potential assets and strengths that the family might possess, which may in turn help buffer the impacts of experiences with poverty on early child outcomes (e.g., Burstein et al., 2021; Han et al., 2023; Longhi et al., 2021; Narayan et al., 2023; Sege et al., 2022). This is largely due to the lack of data related to potential family-, parent-, and/or child-level strengths within our earlier timepoints of data collection. As we continue to analyze data from this longitudinal project, we can re-evaluate the current model and predictive outcomes within our sample by also adding in potential mediator(s) and/or moderator(s) from later timepoints when children are older, such as measures of neighborhood quality, access to resources and social support systems, as well as measures of positive parent-child interactions and parent coping strategies. While not possible with the data presented in the current work, future work should incorporate a strength-based approach to understanding whether other family characteristics may mitigate the effects of economic hardship and pressure on child outcomes when evaluating the Family Stress Model.

Next, as both the key indicators within our Family Stress Model and the child language skills analyzed here are self-reported by mothers (versus observer-rated), this may impact the overall reliability of our findings. The use of such as physiological markers of stress (like hair cortisol concentrations) and/or assessor-rated observations of disrupted parenting, as well as assessor-rated measures of child language skills collected at later timepoints from participants in the SMALL Talk study, may provide a more nuanced picture of the ways in which the Family Stress Model pathway impacts early language outcomes within our sample. Finally, given the longitudinal nature of this study and the involvement of a historically marginalized population, there were missing data and attrition across timepoints.

## 5. Conclusion

The present findings support the hypothesized directional relationship of the Family Stress Model in explaining late talker status around age 2 years in young children growing up in low-income households. Building upon our previously validated Family Stress Model, using 14 key indicators to represent the five key stressors proposed by Masarik and Conger (2017), our analyses suggest a mechanism through which experiences with poverty may disrupt early language development, although further exploration of the impacts of family dynamics within this crucial developmental period is warranted.

Potential implications of this study warrant discussion. First, to our knowledge, the current study is the first effort to comprehensively map the stressors of poverty – *economic hardship, economic pressure, parent psychological distress, interparental relationship problems, and disrupted parenting* – within the early home context for young children and their families, and to consider how these may serve as mechanisms through which experiences with poverty relate to late language emergence. While disrupted parenting has a notable influence on children’s late talker status, this model suggests various aspects of family stressors that should be better understood and potentially intervened upon to decrease the prevalence of language delay and disorder in families experiencing poverty. Additionally, we encourage future studies to attempt to balance measures of potential family strengths and assets with those of stresses and strains within tests of the Family Stress Model in future research samples to better understand how the combination and interplay of both negative *and* positive conditions and experiences come together to shape child outcomes in more nuanced ways during experiences of economic hardship and pressure.

## Figures and Tables

**Table 1:** Description of family stress model measurements.

Dimension of Family Stress Model	Key Indicator	Description of Measurement	Scoring	Internal consistency
Economic Hardship	Income-to-needs ratio	Participants were asked their total annual household income for the previous year, using $10,000 increments (i.e., *$10,000 or less; $10,001-$20,000; $20,001-$30,000; …; $90,000 or more*), as well as how many individuals total lived in their household.	Calculated based on the median reported income range divided by the 2020 poverty line based on household size: higher score = lower hardship	N/a
	Access to health insurance	Participants were asked “what kind of health insurance or health care coverage do you have?” and answered using the following options: *private health insurance or HMO; Medicaid; military health care/TRICARE/CHAMPUS/CHAMPVA; Indian health service; Another government program (e.g., Medicare); Other (please specify);* or *None*.	Ordinal variable, such that participants scored 0 = no insurance, 1 = Medicaid/Government-aided insurance, and 2 = Private or multiple insurance(s): higher score = more access to healthcare resources and less hardship	N/a
	Availability of financial resources	Participants responded *yes* (= 1) or *no* (= 0) to four statements about resources they (or someone in their household) possess, including a (1) checking account, (2) savings account, (3) credit card, and (4) driver’s license (an indicator of having access to obtain household needs, and thus economic hardship; Yoshikawa et al., 2008)	Average score, only for participants who answered all 4 items: higher score = more financial security and less hardship	α = .65~.71
Economic Pressure	Food insecurity	USDA Household Food Security Scale (Bickel et al., 2000) – 18 items assessing family’s ability to meet food needs, responses/scores vary by question (see reference)	Average score, only for participants who answered at least 15 items: higher score = higher food insecurity	α = 0.89~.90
	Household chaos	**CHAOS-SF**: Confusion, Hubbub, and Order Scale (CHAOS; Matheny et al., 1995), short form (first used by Petrill et al. (2004) and then by many others, as shown by Marsh et al. (2020)) – 6 items assessing overall feeling of home atmosphere, rated on 5-point scale (1 = *Definitely untrue* to 5 = *Definitely true*)	Average score, only for participants who answered at least 5 items: higher score = higher level of household chaos experienced in the home	α = .59 (same at both TP1 and TP2)
Parent Psychological Distress	Depression	**CESD-R**: Center for Epidemiological Studies of Depression Scale – Revised Form (Eaton et al., 2004; Van Dam & Earleywine, 2011) – 20 items assessing symptoms of depression over last two weeks, rated on 5-point scale (0 = *Not at all or <1 day* to 4 = *Nearly every day*)	Average score, only for participants who answered at least 16 items: higher score = higher prevalence of depressive symptoms	α = .94 (at TP1 only)
	Anxiety	**GAD-7**: Generalized Anxiety Disorder 7-Item (Löwe et al., 2008; Spitzer et al., 2006) – 7 items assessing symptoms of anxiety over last two weeks, rated on a 4-point scale (0 = *Not at all* to 3 = *Nearly every day*)	Average score, only for participants who answered at least 6 items: higher score = higher prevalence of anxiety symptoms	α = .92~.93
	Stress	**PSS**: Perceived Stress Scale (Cohen, 1994; Cohen et al., 1983) – 10 items assessing mother’s perception of stress experienced in day-to-day life over the last month, rated on a 5-point scale (0 = *Never* to 4 = *Very often*)	Average score, only for participants who answered at least 8 items: higher score = higher prevalence of perceived stress	α = .87 (at TP2 only)
	Experience with discrimination	**EDS**: Everyday Discrimination Scale (Williams et al., 1997) – 9 items assessing mother’s experiences with different topics of discrimination, rated on 6-point scale (0 = *Never* to 5 = *Almost every day*)	Average score, only for participants who answered at least 8 items: higher score = higher perceived experience with discrimination	α = .89 (at TP2 only)
Relationship Problems	Family conflict	**FES**: Family Environment Scale – Conflict Subscale (Moos, 1974; Moos & Moos, 2009) – 9 items assessing negative communication and conflict in the family, rated as *true* (= 1) or *false* (= 0)	Dichotomized scores (due to skewed nature of data), such that 1 = at least one statement out of 8 items indicated as *true* and 0 = false to all items: higher score = higher conflict	α = .70~.77
	Relationship conflict	Shortened version of Braiker-Kelley Relational Intimacy Scale – Conflict-Negativity Subscale (Braiker & Kelley, 1979)– 4 items (the first four of the original 5-item scale) assessing behavioral conflict and negative communication, rated on 5-point scale (1 = *Never* to 5 = *Always*)	Average score, only for participants who answered all 4 items: higher score = higher conflict	α = .65~.66
Disrupted Parenting	Parenting self-efficacy	**PACOTIS**: Parental Cognitions and Conduct Toward the Infant Scale – Parental Self-Efficacy Subscale (Boivin et al., 2005) – 6 items assessing mother’s feelings of effectiveness as a parent, rated on 11-point scale (0 = *Not at all true* to 10 = *Completely true*)	Average score, only for participants who answered at least 5 items: higher score = higher perceived effectiveness	α = .78~.87
	Mother-child attachment	**MPAQ**: Maternal Postnatal Attachment Questionnaire – Quality of Attachment Subscale (Condon & Corkindale, 1998) – 9 items assessing quality of attachment between mother and child, responses/scores vary by question (see reference)	Average score, only for participants who answered at least 8 items: higher score = higher quality attachment	α = .53~.73
	Parenting Aggravation	Fragile Families Aggravation in Parenting Questions (The Fragile Families and Child Wellbeing Survey, 2005) – 9 items assessing how parenting makes the mother feel, rated on 4-point scale (1 = *Strongly agree* to 4 = *Strongly disagree*)	Average score, only for participants who answered at least 8 items: higher score = lower aggravation in parenting	α = .72~.75

**Table 2:** Description of the analytical sample (*n* = 246).

	Valid *N*	% or Mean	*SD*	Min	Max
*Child Demographics*					
Child age in months					
TP1	227	8.54	1.59	5	14
TP2	235	14.34	1.12	11	19
TP3	236	19.94	1.26	18	25
TP4	213	25.52	2.06	22	32
Child gender: Female	246	47 %			
Child race	234				
White		31 %			
Black/African American		43 %			
Other		6 %			
Multiracial		20 %			
Child ethnicity: Hispanic	235	11 %			
*Mother and Family Demographics*					
Mother age in years (year 1)	244	29.26	5.52	19	42
Mother race	242				
White		41 %			
Black/African American		43 %			
Other		9 %			
Multiracial		7 %			
Mother ethnicity: Hispanic	244	7 %			
Mother’s highest level of education	244				
Less than high school		12 %			
High school diploma or GED		27 %			
Some college or two-year degree		40 %			
Bachelor’s degree or higher		21 %			
Mother’s marital status	243				
Married and living with a partner		33 %			
Not married but living with a partner		22 %			
Not living with a partner		45 %			
Annual household income (in $1000)	239	26.53	17.57	5	95
Number of people in household	246	4.50	1.55	2	11
Primary home language: English	246	87 %			
*Child Language Skills*					
CDI-WG Words Produced Percentile Rank (TP2)	199	40.40	21.79	5	99
CDI-WS Words Produced Percentile Rank (TP3)	233	37.07	29.46	5	99
CDI-WS Words Produced Percentile Rank (TP4)	196	40.56	32.54	5	99
CDI-WS Late Talker status (TP3)	233	29 %			
CDI-WS Late Talker status (TP4)	196	30 %			

Note. For Late Talker status, 1 = Identified as a late talker, and 0 = Not identified as a late talker.

**Table 3:** Bivariate correlations among study variables.

	1	2	3	4	5	6	7	8	9	10	11	12
1. Economic Hardship (factor score)	1.00											
2. Economic Pressure (factor score)	.31	1.00										
3. Parent Psychological Distress (factor score)	.12	**.87**	1.00									
4. Relationship Problems (factor score)	−.01	**.72**	**.82**	1.00								
5. Disrupted Parenting (factor score)	−.04	**.67**	**.83**	**.84**	1.00							
6. CDI-WS Late Talker status (TP3)	−.03	−.07	−.02	−.02	−.03	1.00						
7. CDI-WS Late Talker status (TP4)	.08	.03	−.04	−.03	−.00	**.66**	1.00					
8. Child’s gender (0 = male, 1 = female)	.03	.04	.07	.00	−.01	.04	−.04	1.00				
9. Child’s age in months (TP3)	.04	.12	.17	.15	.15	.05	−.03	−.09	1.00			
10. Child’s age in months (TP4)	−.06	.02	.05	.05	.02	−.05	−.07	−.10	.28	1.00		
11. Home language (0 = English, 1 = other language)	−.04	**−.14**	**−.16**	−.07	−.05	.04	.10	−.04	.01	.02	1.00	
12. CDI-WG Words Produced percentile rank (TP2)	−.01	−.05	−.11	−.12	−.09	**−.61**	−.40	−.02	−.01	−.06	−.02	1.00

Note: For Late Talker status, 1 = Identified as a late talker, and 0 = Not identified as a late talker. Pearson correlation coefficients were calculated for correlations between two continuous variables, two dichotomous variables, or a continuous variable and a dichotomous variable. Spearman correlation coefficients were calculated for correlations between two ordinal variables, or between an ordinal variable and a continuous/dichotomous variable. Strong correlations are emphasized in **bold** font.

**Table 4:** Comparison of family stress variables and covariates for children with Late Language Emergence (LLE) and peers (non-LLE).

	LLE				Non-LLE			
	Valid *N*	% or Mean	*SD*	Range	Valid *N*	% or Mean	*SD*	Range
Family Stress Variables								
Economic Hardship	58	.04	1.06	−3.61~1.52	138	−.13	.91	−2.34~1.74
Economic Pressure	58	−.00	.95	−1.44~2.08	138	−.06	.94	−1.57~2.72
Parent Psychological Distress	58	.04	.99	−1.52~2.87	138	−.06	.96	−1.60~3.18
Relationship Problems	58	−.05	.96	−1.75~2.51	138	.00	.93	−2.11~2.13
Disrupted Parenting	58	−.02	.96	−1.49~3.09	138	−.02	.96	−1.55~2.95
Covariates								
Child is a girl	58	43 %			138	47 %		
Child primary home language is non-English	58	19 %			138	12 %		
Child Late Talker status (TP3)	55	73 %			128	9 %		
Child age in months (TP4)	58	25.16	1.79	22~30	138	25.42	1.82	22~30
CDI Words Produced percentile rank (TP2)	48	27.92	17.07	5~75	114	46.18	20.95	5~99

*Note*. Late language Emergence status is assessed at TP4.

**Table 5:** Family stressors and early language disruption: Results of the path model (*n* = 165).

Pathway	*β*	*SE*	*P*
Economic Hardship → Economic Pressure	0.19	0.10	0.042
Economic Pressure → Parent Psychological Distress	0.85	0.04	<0.001
Parent Psychological Distress → Relationship Problems	0.85	0.06	<0.001
Parent Psychological Distress → Disrupted Parenting	0.53	0.06	<0.001
Relationship Problems → Disrupted Parenting	0.36	0.07	<0.001
Disrupted Parenting → Child Late Talker status (TP3)	−0.18	0.14	0.188
Disrupted Parenting → Child Late Talker status (TP4)	0.74	0.63	0.241
Control Variables for Child Late Talker Status (TP4)			
Child gender (0 = male, 1 = female)	−0.50	0.69	0.465
Child age (TP4)	−0.26	0.39	0.504
Child primary home language is non-English	0.32	1.12	0.778
Child Late Talker status (TP3)	2.57	1.44	0.074
Language pre-test (CDI Words Produced percentile rank TP2)	1.05	0.85	0.217
Control Variables Child Late Talker Status (TP3)			
Child gender (0 = male, 1 = female)	0.11	0.30	0.709
Child age (TP3)	0.08	0.10	0.405
Child primary home language is non-English	0.33	0.42	0.433
Language pre-test (CDI Words Produced percentile rank TP2)	−1.13	0.14	<0.001

Note: For Late Talker status, 1 = Identified as a late talker, and 0 = Not identified as a late talker.

*β* represents unstandardized coefficient. All continuous variables were standardized to have a mean of 0 and standard deviation of 1.

## Data Availability

The datasets generated during and/or analyzed during the current study are available from the corresponding author upon reasonable request.
